# Climate Change May Expand Geographic Distribution of Asian Butterflies Despite Climatic Niche Contraction

**DOI:** 10.3390/insects17070683

**Published:** 2026-07-01

**Authors:** Ehsan Rahimi, Chuleui Jung

**Affiliations:** 1 Agricultural Research Institute, Gyeongkuk National University, Andong 36729, Republic of Korea; ehsanrahimi666@gmail.com; 2 Department of Plant Medicasl, Gyeongkuk National University, Andong 36729, Republic of Korea

**Keywords:** niche contiguity, niche ghost, pollinators, insects, species distribution modeling

## Abstract

This study presents a nuanced understanding of how butterflies may respond to climate change, suggesting that although many species could experience noticeable contractions in their climatic niches, a substantial number may still have the potential to considerably expand their geographic ranges. We modeled the distributions of 200 butterfly species across Asia using the MaxEnt algorithm. Habitat suitability maps were produced for both current conditions and future scenarios under moderate (SSP245) and high (SSP585) greenhouse gas emissions. To analyze niche dynamics, we projected the realized niches of species onto principal component environmental spaces, which allowed us to quantify key processes including niche stability, expansion, unfilling, abandonment, and pioneering. These indices were then mapped spatially to identify potential changes in climatic niche configurations and geographic distributions in response to climate change. Our findings indicate that by 2070, under the moderate-emissions scenario (SSP245), 185 species are projected to show expansion in suitable habitat by an average of 38%, while 15 species may face an average decline of 11%. Under the high-emissions scenario (SSP585), 184 species are projected to show increases in suitable habitat by 55% on average, with 16 species showing an average decrease of 13%. Additionally, species are predicted to maintain approximately 63% of their current climatic niche stability under SSP585. Niche expansion averages 12%, reflecting potential range growth, whereas unfilling accounts for around 16%, indicating possible loss of some currently suitable areas. Niche abandonment or niche contraction, representing climatic spaces no longer projected to be occupied, comprises about 8%, and pioneering into entirely new climatic conditions remains limited at roughly 1%. The modeled presence of niche unfilling and abandonment suggests that butterflies may retain considerable ecological flexibility, potentially allowing them to shift into new environments under changing climate conditions. This projected adaptability may enhance their resilience by facilitating potential movement into novel or previously unoccupied habitats, though actual range shifts will depend on factors beyond climatic suitability alone.

## Simple Summary

This study examined how 200 butterfly species across Asia might respond to climate change by modeling their current and future habitat suitability. Using two emissions scenarios (moderate and high) projected to 2070, the researchers found that the majority of species—around 184–185 out of 200—are expected to expand their suitable habitats significantly (by 38–55% on average), while only a small number face decline. Butterflies are also predicted to retain roughly 63% of their current climatic niche stability. Overall, the findings suggest that Asian butterflies are ecologically flexible enough to adapt to shifting climate conditions, allowing many species to colonize new habitats and maintain resilience despite ongoing climate change.

## 1. Introduction

Butterflies, with more than 19,000 extant species worldwide, represent one of the most intensively studied insect groups, owing to their rich natural history records and their long-standing role in ecological and evolutionary research [1]. Global analyses reveal that centres of butterfly richness, range rarity, and phylogenetic diversity—covering 12,119 species—are disproportionately concentrated in tropical and subtropical mountain systems [2]. These insects play critical ecological roles: as larvae, they function as herbivores, and as adults, they serve as pollinators, including for 54% of the world’s food crops [3]. Unlike many taxa whose abundance patterns largely track vegetation change, butterflies respond rapidly to environmental shifts, making them valuable bioindicators [4]. Their widespread popularity and appeal as model organisms have further reinforced their scientific and public importance [5].

Despite their ecological and cultural significance, many butterfly populations are in steep decline. Long-term monitoring in the UK, the Netherlands, and Belgium reveals severe downward trends, while across 16 European nations, grassland butterfly populations have dropped by 39% since 1990 [6]. The 2010 European Red List reports that 8% of species are threatened and 10% are near threatened, with central and mid-western Europe facing the highest risk. Habitat loss, degradation, and chemical pollution remain dominant drivers, and climate change is both shifting species’ ranges northward and introducing new threats [6]. Addressing these declines demands integrated conservation actions, including habitat protection, mitigation of chemical pollutants, and the implementation of policy reforms to safeguard butterfly diversity and the ecological services they support.

Understanding how climate change will affect butterflies requires a detailed examination of the multiple dimensions of their climatic niches. Niche dynamics can occur in both analogue and non-analogue climatic spaces, each with distinct implications for predicting species’ responses to environmental change [7,8,9]. In non-analogue spaces—climates that are absent in either the current or projected future range—two processes are recognised: niche abandonment, where climates once occupied under current conditions are no longer represented in the future, and niche pioneering, where a species is projected to occupy entirely novel climatic conditions with no historical precedent within its range [10].

In analogue climatic spaces—shared between native and introduced ranges—three additional processes shape niche dynamics: unfilling, expansion, and stability [10]. Unfilling occurs when climates present in the current range are no longer occupied under future conditions, potentially reflecting the loss of currently suitable areas as climate shifts beyond what species currently experience [11]. Expansion describes the projected occupation of climates in the future range that are currently absent, potentially arising from shifts in fundamental niche limits or changes in biotic interactions such as release from competitors or natural enemies [12]. Stability reflects climatic conditions that remain consistently occupied across both current and future ranges.

A related and increasingly recognised concept is that of niche truncation, which occurs when a species’ observed ecological niche—estimated from its current distribution—does not fully represent its fundamental niche because portions of that niche lie beyond the boundaries of climates that currently exist on Earth [13,14]. Where a species’ niche limits are contiguous with the outer boundaries of presently available climatic conditions, climate change may effectively unlock sections of the fundamental niche that are currently inaccessible. This phenomenon, referred to as niche contiguity, reflects the boundary between a species’ realised ecological niche and adjacent portions of its fundamental niche that could potentially be colonised if suitable climatic conditions emerge in the future and dispersal or biotic constraints do not prevent establishment [13]. The theoretical basis for such pre-adaptation rests on ecological niche conservatism—the tendency of species to retain ancestral climatic tolerances [15]—and on the fact that many taxa evolved under warmer conditions than those that prevail today, meaning their actual thermal tolerance limits may extend beyond what current distributions suggest [16,17].

Chevalier, Broennimann and Guisan [13] quantified the prevalence of niche truncation at a global scale across 24,944 terrestrial and marine species, finding that nearly half (~49%) exhibited niche contiguity, with the phenomenon especially pronounced in tropical ecosystems. Using niche expansion scenarios, they estimated that 86% of species showing contiguity could potentially occupy climates beyond their current limits under future warming, leading to lower—though still concerning—predicted rates of biodiversity loss, particularly in the tropics. These findings have important implications for butterfly distribution modelling, as many Asian butterfly species inhabit warm tropical environments where niche truncation is most likely to occur, and where the emergence of novel climatic conditions under high-emissions scenarios may open portions of the fundamental niche that are presently unreachable.

Tropical Asia—here broadly encompassing South and Southeast Asia—represents one of the most biologically diverse yet highly threatened regions on Earth. The region’s complex geological history, shaped by repeated connections and separations of over 20,000 islands during dramatic sea-level changes, has fostered the evolution of many species restricted to single islands or island groups [14]. This dynamic past has produced extraordinary levels of endemism, with the region harbouring an estimated 15–25% of all well-studied terrestrial taxa, alongside a substantial proportion of yet undescribed species [15]. However, the same area faces severe conservation challenges: projections indicate that 42% of Southeast Asia’s biodiversity could disappear by 2100, driven largely by the loss of three-quarters of its primary forests to agriculture, urbanisation, and mineral extraction [16,17].

In this study, we investigate how climate change could alter the future distributions of 200 butterfly species across Asia, with a particular focus on changes in their climatic niche structure. Specifically, we quantify shifts in niche abandonment, pioneering, expansion, unfilling, and stability. Here, niche abandonment corresponds to the “niche ghost” concept—future contractions into climatic spaces for which species remain pre-adapted. While most previous research has examined these processes in invasive species, our approach is novel in applying niche dynamic analysis to a wide set of native species under both current and projected climate scenarios. This dual perspective not only provides insights into the vulnerability of Asia’s butterflies but also informs conservation strategies in one of the planet’s most critical biodiversity hotspots.

## 2. Methodology

### 2.1. Study Area

**Figure 1:**
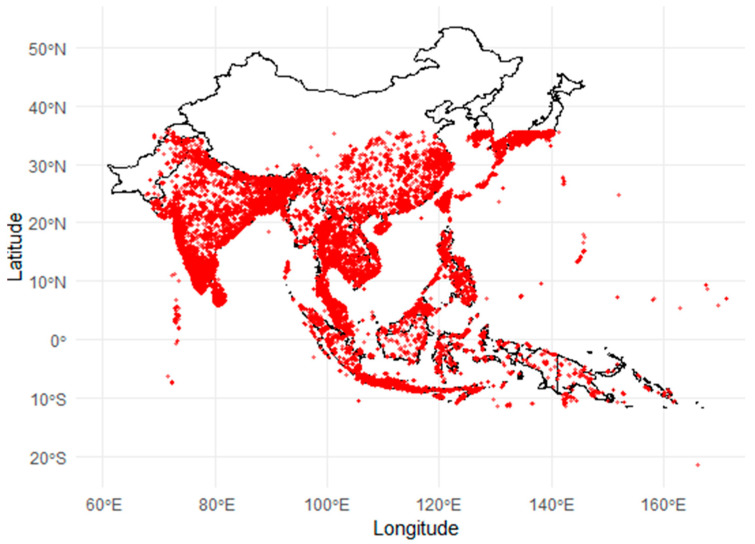
Study area map showing the geographical extent of the Asia shapefile used in this study, overlaid with all butterfly occurrence points compiled from 200 species across multiple data sources. Points represent recorded species presence locations before spatial filtering was applied to reduce sampling bias.

Our study focuses on Asia, with a particular emphasis on tropical Asia and Southeast Asia, as illustrated in Figure 1. The map highlights the study area boundary, overlaid with red dots representing the occurrence records of 200 butterfly species collected within this region. These points reflect spatially filtered presence data used for subsequent species distribution and niche dynamic analyses.

### 2.2. Data

Despite recent advances in mapping butterfly diversity, significant gaps remain in global datasets [18,19]. Although several studies have produced global butterfly distribution maps [20,21,22], species distribution modelling (SDM) for butterflies is still challenged by taxonomic complexities, particularly the prevalence of synonyms. Many researchers rely heavily on GBIF data; however, for butterflies, synonymy can reduce the reliability of raw occurrence records. To address this, we used the comprehensive dataset compiled by Yau et al. [23], which integrates 730,190 occurrence records for 3752 tropical Asian butterfly species from multiple sources: GBIF (651,285 records), published literature (27,217), published databases (37,695), and unpublished datasets (13,993). The 200 butterfly species modeled in this study belonged to six families, with Nymphalidae representing the largest proportion (89 species), followed by Lycaenidae (46), Papilionidae (23), Hesperiidae (22), Pieridae (18), and Riodinidae (2). This taxonomic composition reflects the dominant butterfly families of tropical and subtropical Asia, with Nymphalidae and Lycaenidae together accounting for more than two-thirds of the total species included in the analysis.

**Figure 2:**
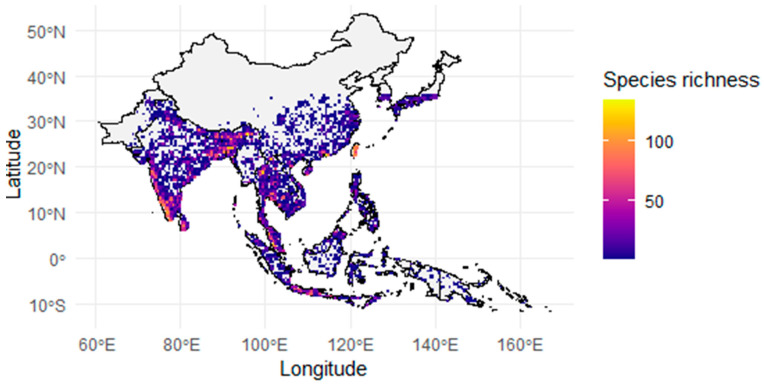
Species richness map of butterflies in Asia derived from occurrence data aggregated at a 0.5° grid resolution. The raster cells represent the number of unique butterfly species recorded within each grid cell, highlighting butterfly hotspots concentrated in tropical and subtropical regions of the study area.

The Yau, Jones, Tsang, Xing, Corlett, Roehrdanz, Lohman, Lee, Hai and Chowdhury [23] dataset is taxonomically verified, cleaned, and spatially harmonised, providing a robust foundation for large-scale ecological analyses. It also includes single-species distribution maps for 1576 species, offering a valuable reference for biodiversity modelling in the region. For this study, we selected the top 200 species (Figure 2) with the highest number of occurrence records to ensure robust modelling performance. To reduce spatial sampling bias and limit the spatial autocorrelation introduced by clustered sampling, we applied a 2-cellsize (~9 km) minimum distance filter between occurrence points using the *flexsdm* package [24] in R package version 1.4.0, thereby retaining only spatially independent records suitable for reliable SDM calibration.

### 2.3. Environmental Variables

In this study, we obtained 19 bioclimatic variables—comprising 11 temperature and 8 precipitation metrics—from the WorldClim database (www.worldclim.org, accessed on 10 February 2026). Because such predictors often exhibit strong multicollinearity, we used the *usdm* package version: 2.1-7 [25] to conduct a Variance Inflation Factor (VIF) analysis and retain only non-correlated variables for each continent. This process resulted in a final subset of six predictors: BIO3 (Isothermality), BIO4 (Temperature Seasonality), BIO13 (Precipitation of Wettest Month), BIO14 (Precipitation of Driest Month), BIO18 (Precipitation of Warmest Quarter), and BIO19 (Precipitation of Coldest Quarter), representing a balanced combination of temperature- and precipitation-related climate descriptors. For future projections, we adopted two shared socioeconomic pathways: SSP245, a moderate-emissions scenario, and SSP585, a high-emissions trajectory associated with an estimated global mean temperature increase of 4.4 °C by 2070 [26]. Future climate data were derived from a single General Circulation Model (GCM), specifically the ACCESS-CM2 model (Australian Community Climate and Earth System Simulator, Coupled Model version 2), obtained from the WorldClim database at a spatial resolution of 2.5 arc-minutes.

### 2.4. Model Fitting

Species distribution modelling was conducted using the *flexsdm* R package version 1.4.0 [24], implementing the MaxEnt algorithm to generate climate suitability maps from species presence data and selected bioclimatic predictors. To improve model calibration, we supplemented the occurrence dataset with 10,000 randomly generated pseudo-absence points restricted to the study area. Model evaluation was based on three complementary performance metrics provided within *flexsdm*: the inverse mean absolute error (IMAE) [27], the area under the receiver operating characteristic curve (AUC) [28], and the Boyce Index (BOYCE) [29]. AUC values between 0.7 and 0.9 were interpreted as indicating good predictive performance, while values above 0.9 denoted excellent accuracy. IMAE, calculated as 1 − Mean Absolute Error, follows the same logic, with higher values representing stronger model performance.

The Boyce Index evaluates the correlation between predicted suitability and the distribution of observed presences, where values approaching 1 indicate high predictive power, values near 0 reflect random predictions, and negative values suggest poor performance. All models were validated using a 5-fold cross-validation framework [30] based on random partitioning. We note that random partitioning does not fully enforce spatial independence between training and testing data; the prior spatial thinning step (Section 2.2) was applied in part to mitigate this. We generated binary suitability maps for both current and future climate scenarios of SSP585 by applying a threshold of 0.6 to the continuous habitat suitability values [31]. The threshold of 0.6 was selected to retain only areas of relatively high predicted habitat suitability, thereby minimizing the inclusion of marginally suitable or false-positive predictions while maintaining an ecologically meaningful binary representation of species distributions.

### 2.5. Quantification of Niche Dynamics

The input data for the niche dynamics analysis consisted of the entire study area, where each raster cell represented a geographic location with associated bioclimatic variable values. For both the current climate and the future SSP585 scenario, bioclimatic values were extracted for every cell across the study region. These data were combined into matrices representing the environmental background, effectively characterizing the fundamental niche space available to all species. A principal component analysis (PCA) was then performed separately for current and future conditions to reduce the dimensionality of the environmental variables and to generate comparable ordination spaces representing the fundamental climatic niche.

In the next step, for each species, we extracted raster cell values from binary habitat suitability maps under both current and future SSP585 scenarios. We applied a threshold of 0.6 to continuous suitability values, designating cells above this cutoff as highly suitable and representative of the species’ realized niche in each scenario. These realized niche points were then projected onto their corresponding fundamental niche PCA spaces, allowing visualization and quantification of niche overlap, expansion, contraction, and shifts between current and future periods. This approach enabled a direct comparison of how species’ realized climatic niches may change relative to the broader environmental space, providing insights into potential niche stability and dynamics under climate change.

These data were then projected onto the PCA environmental space using the ecospat package version 4.1.4 [32] function suprow, which performs the projection of supplementary data onto an existing PCA ordination. This approach allowed us to represent species niches as points in the same reduced environmental space, facilitating direct comparison between current and future niche distributions. We quantified niche dynamics using the ecospat function *ecospat.niche.dyn.index*, which computes key indices describing niche stability, expansion, unfilling, pioneering, and abandonment by comparing kernel-smoothed climatic niche grids derived for current and future periods (*ecospat.grid.clim.dyn*).

These indices characterize the degree to which species maintain, lose, or gain climatic niche space under climate change. To ensure data quality, we applied filtering steps to remove missing or outlier values and confined analyses to the environmental background space defined by the PCA scores of the study region. To visualize spatial patterns of niche changes, we projected the niche dynamic indices from environmental space back to geographic space using the ecospat function *ecospat.niche.dynIndexProjGeo*. This function leverages the environmental raster stack—a multi-layered spatial dataset in which each layer represents a single climatic variable aligned across identical grid cells and spatial extents—to map the environmental PCA grid cells onto geographic coordinates, producing spatially explicit raster maps of niche dynamics for each species. Unlike a single raster map, which displays the spatial distribution of only one variable at a time, the raster stack integrates multiple climatic layers simultaneously, enabling the simultaneous extraction and projection of multivariate environmental conditions across geographic space.

## 3. Results

### 3.1. Model Assessment

Table 1 summarizes the model validation metrics for species distribution models generated using the MaxEnt algorithm across 200 butterfly species in Asia. The high mean AUC value of 0.92 (±0.01) reflects an excellent overall model discrimination ability between presence and background locations. Similarly, the Boyce Index averaged 0.92 (±0.03), demonstrating strong agreement between predicted suitability and observed occurrences, which confirms the models’ reliability in predicting habitat suitability. The IMAE value of 0.87 (±0.04) further supports the high predictive accuracy of the models, indicating low error in suitability estimates.

### 3.2. Climate Change Effects on Geographic Distribution

Table 2 presents the average percentage changes in the area classified as highly suitable habitat for butterfly species in Asia under two climate change scenarios projected for 2070: SSP245 and SSP585. The table reports both the number of species expected to experience increases or decreases in their suitable habitat areas and the corresponding average percentage changes, with standard deviations provided in parentheses to indicate variability among species. Under the moderate-emission scenario SSP245, 185 species are predicted to have an increase in suitable areas, averaging a 37.6% expansion (±22%), while 15 species are projected to experience a decrease, averaging an 11.4% reduction (±9%). Under the high-emission SSP585 scenario, the number of species with increasing suitable habitat remains similar (184 species), but the average expansion is notably larger at 54.9% (±31%), whereas 16 species are expected to decline, with an average decrease of 13.4% (±11%).

**Figure 3:**
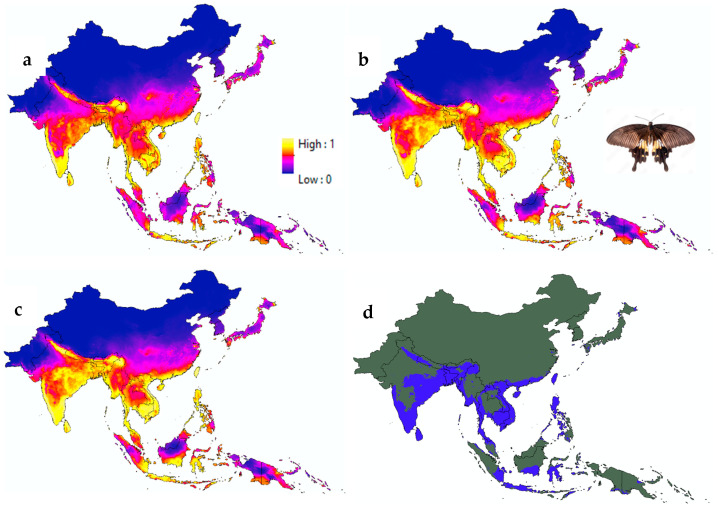
Habitat suitability maps of Papilio polytes under different climate scenarios: (**a**) current, (**b**) future projection under SSP245, (**c**) future projection under SSP585, and (**d**) binary suitability map for SSP585. In the continuous suitability maps (**a**–**c**), blue indicates areas of low habitat suitability, while yellow represents areas of highest suitability. In the binary map (**d**), blue denotes suitable habitat (values above the threshold), and dark green indicates unsuitable areas.

Figure 3 presents suitability maps for *Papilio polytes* across various climate scenarios: (a) current conditions, (b) future projections under SSP245, (c) future projections under SSP585, and (d) a binary suitability map for SSP585. The maps indicate that, under climate change, eastern and northern regions of India, as well as southern parts of Indonesia and Thailand, are likely to experience an increase in habitat suitability and potential species distribution. Overall, this figure illustrates a range expansion of the selected example species in response to future climatic shifts.

Detailed information on all species is provided in the Data Availability Statement Section. According to these data, *Iraota timoleon* exhibits the greatest potential habitat increase, with gains of 134.4% under the SSP2-4.5 scenario and an even larger 190.7% under SSP5-8.5. *Delias eucharis* follows closely, with increases of 104.3% and 128.3% in the respective scenarios. Other notable expansions include *Kallima inachus* (85.2% and 115.2%), *Udaspes folus* (84.7% and 119.3%), and *Telicota bambusae* (80.7% and 113.0%). Species such as *Euploea core* (78.3% and 107.9%), *Cepora nerissa* (77.8% and 112.7%), and *Papilio polymnestor* (76.8% and 84.9%) also show significant projected growth. *Tirumala limniace* (75.4% and 113.2%) and *Rapala manea* (75.3% and 107.6%) complete the top ten, with all showing greater gains under the high-emissions SSP5-8.5 scenario. These projections suggest that generalist or environmentally tolerant species may expand their range considerably in the coming decades, particularly under stronger climate change conditions.

Among the butterfly species projected to experience the steepest declines in suitable habitat, *Hestina assimilis* faces the most dramatic reductions, with losses of 30.6% under SSP2-4.5 and 44.1% under SSP5-8.5. *Faunis eumeus* also shows a substantial decrease, particularly under SSP5-8.5 (−28.1%). *Papilio bianor* is projected to lose nearly a quarter of its current range under SSP2-4.5 (−24.6%) and more than a quarter under SSP5-8.5 (−27.1%). *Dryas iulia* follows closely, with declines of 24.5% and 20.4% for the two scenarios. Other species with notable reductions include *Neope muirheadii* (−14.2% and −13.8%), *Parantica agleoides* (−15.1% and −13.6%), *Eooxylides tharis* (−15.9% and −12.7%), and *Curetis acuta* (−2.6% and −10.5%). While *Polygonia caureum* shows a small gain under SSP2-4.5 (5.1%), it is expected to decline by 10.0% under SSP5-8.5. *Limenitis sulpitia* also shows modest losses in both scenarios (−8.1% and −7.9%). These patterns suggest that more specialized or habitat-restricted species may be especially vulnerable to climate change, particularly under high-emissions futures.

### 3.3. Niche Dynamics Under Climate Change

**Figure 4:**
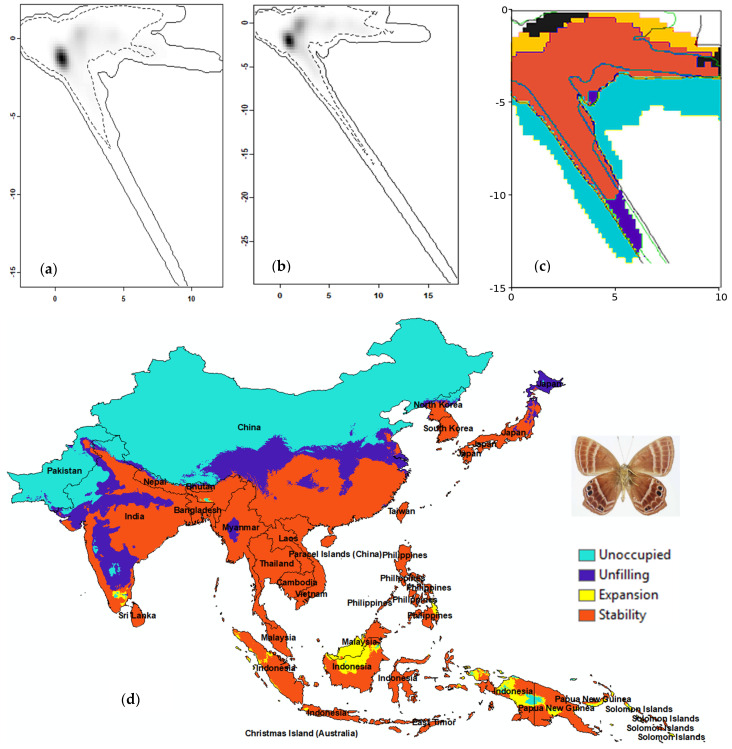
Climatic niche of *Abisara echeria* shown for (**a**) current conditions and (**b**) future projections under the SSP585 scenario for 2070. Panel (**c**) depicts niche overlap, illustrating niche stability (red), niche expansion (yellow), and niche unfilling (blue). Solid contour lines represent the full extent of environmental conditions shared between native and invaded ranges, while dotted contour lines indicate the quantile threshold used in the analysis. In panel (**d**), the projected niche dynamics are mapped onto geographic space, where yellow areas represent niche expansion and red areas indicate niche stability.

Figure 4 illustrates the climatic niche of *Abisara echeria* as a representative example to explain the results of this study. Figure 4a,b display the species’ climatic niche in current and future conditions (SSP585 scenario for 2070), respectively, visualized within a PCA environmental space. Figure 4d projects the niche overlap geographically onto a map of Asia, revealing that most of the species’ niche is expected to remain stable in the future. However, northern parts of its range may become unoccupied, indicating potential for future colonization if dispersal barriers are removed. Additionally, the species may expand into central regions of Indonesia, suggesting an overall range expansion toward southern Asia. Using the ecospat function ecospat.niche.dyn.index, we quantified niche dynamics—including pioneering, expansion, stability, unfilling, and abandonment—in terms of the number of raster cells occupied. This analysis was conducted for all 200 species in the study, with detailed results and niche overlap plots provided in the Data Availability Statement Section.

Table 3 summarizes key aspects of climatic niche dynamics for 200 butterfly species facing future climate conditions under the high-emission SSP585 scenario. On average, stability constitutes the largest portion of niche dynamics, with approximately 62.8% (±15%) of species’ climatic niches remaining consistent between current and future conditions. This indicates that most of the environmental conditions currently occupied by these species are expected to persist, suggesting a substantial degree of niche conservatism and potential persistence in core suitable habitats despite climatic shifts. Expansion represents the second most prominent category, averaging 12.1% (±10%), reflecting that many species may occupy additional suitable climatic space in the future (shared in native range). This expansion could result from the availability of novel or previously inaccessible climatic conditions, allowing species to extend their realized niche. Such expansions are important for species’ adaptive capacity and range shifts, particularly in regions where warming creates new opportunities for colonization.

The unfilling category, averaging 15.7% (±10%), denotes portions of the current niche that are predicted to no longer be suitable under future climate scenarios. This loss of suitable habitat may reflect range contractions or local extinctions if species are unable to track shifting climates. The relatively high standard deviation highlights interspecific variability, where some species may experience substantial niche loss, while others remain largely unaffected. Abandonment, at 8% (±10%), captures niche space currently suitable but projected to become unsuitable in the future. This metric complements unfilling and may relate to the concept of “niche ghosts” (due to niche contraction) or where climatic conditions once occupied become inaccessible or disappear, potentially challenging species persistence in marginal habitats.

Finally, pioneering accounts for a small fraction (1.1% ± 1%) of niche dynamics, representing colonization of entirely novel climatic conditions not previously occupied. While limited in magnitude, pioneering is ecologically significant as it signals the potential for species to exploit new environmental spaces, though it may also introduce uncertainties in predicting future distributions. Overall, the niche dynamic patterns revealed here suggest that while the majority of butterfly species in Asia may retain stable climatic niches, substantial portions of their realized niches are expected to change. Expansion and contraction processes indicate ongoing range shifts driven by climate change, emphasizing the need for dynamic conservation strategies that account for both stable refugia and emerging suitable areas.

## 4. Discussion

Our modeling of 200 butterfly species shows that climate change is likely to cause substantial shifts in their geographic ranges across Asia by 2070. Range expansion—defined as an increase in suitable habitat compared to the species’ current distribution—was the dominant trend for most species. Under both the moderate-emissions scenario (SSP245) and the high-emissions scenario (SSP585), roughly 92% of species are projected to expand their ranges, with the extent of expansion being greater under SSP585. Notably, in the most extreme pathway, the average projected expansion exceeds 50% of the species’ current geographic extent. This suggests that, under projected climate conditions, suitable habitat may become available in new areas for the majority of butterfly species in Asia, though whether species will successfully colonize these areas depends on dispersal ability, biotic interactions, and other ecological factors beyond climatic suitability alone.

In contrast, findings from other regional studies vary. For example, Rahimi and Jung [33] used SDMs to assess climate change impacts on 47 butterfly species in the Republic of Korea. Under the SSP585 scenario, the analysis revealed that 15 species were projected to expand their ranges by an average of 157%, whereas 32 species were expected to contract by an average of −89%. These results highlight that climate change impacts on butterflies are highly species-specific, with outcomes ranging from substantial expansions to severe contractions, depending on each species’ ecological traits and environmental tolerances.

We assessed niche dynamics to determine whether range expansions under future climate scenarios were accompanied by significant shifts in climatic niches. Niche Stability—defined as the proportion of a species’ current suitable climatic space that remains suitable in the future—emerged as the dominant component of niche dynamics. It averaged 62.8% (±15%) across species, indicating that most species retain most of their present climatic niche. This high stability reflects strong niche conservatism, meaning that the fundamental climatic requirements of these species are likely to persist despite projected climate changes. Niche Expansion—the proportion of future suitable climatic space that is newly available but still falls within the species’ existing climatic tolerance—averaged 12.1% (±10%). This suggests that, in addition to retaining much of their current niche, many species may potentially shift toward newly favorable climatic areas near or within their existing range boundaries, though actual range shifts remain contingent on dispersal capacity and habitat connectivity.

Unfilling—the fraction of the current niche that becomes unsuitable in the future—was 15.7% (±10%) on average. This indicates potential range contraction for many species, with some climatic conditions no longer available in their projected ranges [34]. Niche abandonment—also referred to as niche contraction—describes the loss of climatic conditions that a species previously occupied in its native range but that are absent in its introduced range. In ecological terms, this process can leave behind “niche ghosts,” meaning remnants in the species’ climatic memory that could be recolonized if favorable conditions reappear.

This concept was highlighted by Chevalier, Broennimann and Guisan [13], who addressed its relevance in the context of climate change and questioned the traditionally negative view of such contractions, noting that they may not be permanent losses. In the present study, niche abandonment accounted for 8.0% (±10%), suggesting a measurable reduction in suitable habitat, particularly in marginal or peripheral climatic zones where favorable environments have disappeared completely. Niche Pioneering—colonization of entirely novel climatic conditions not present in the current niche—was the smallest category, averaging 1.1% (±1%). Pioneering is ecologically noteworthy, suggesting that a small subset of species may be projected to encounter entirely novel climatic conditions, though whether this reflects true adaptive capacity requires empirical validation beyond model projections.

Empirical studies related to the impacts of climate change and environmental factors on butterflies generally indicate a decline in butterfly populations and diversity linked to climate change and environmental shifts. For example, Engelhardt et al. [35] reported a significant long-term decrease in butterfly populations across Germany over the past 40 years, reflecting a persistent downward trend. Similarly, Forister et al. [36] observed a notable reduction in species richness for 169 butterfly species at low elevations in California over 35 years. Research by Crossley et al. [37] highlights that changes in precipitation and temperature over the last 26 years have caused butterfly abundance to decrease in hot and dry regions while increasing in cooler, more humid areas. Dar et al. [38] also found that rising temperatures in India negatively affect butterfly abundance and diversity at lower altitudes, prompting shifts toward higher elevations. This pattern of range shifts is further supported by findings in Europe, where future projections suggest a northward movement of butterfly distributions. Additionally, Kukkonen et al. [39] documented a decline in a specific butterfly species population on southern Finnish islands, potentially driven by climate change or other environmental pressures.

## 5. Limitations

Several limitations of the present study should be acknowledged. First, future climate projections relied on a single General Circulation Model (ACCESS-CM2), which may not fully capture the range of possible climate trajectories; the use of multiple GCMs would provide more robust uncertainty estimates and is recommended for future research. Second, although occurrence records were spatially thinned to a 9 km minimum distance, some spatial autocorrelation may remain. Because cross-validation used random rather than spatially blocked folds, model performance metrics (AUC, IMAE, and Boyce) may be somewhat optimistic. Therefore, we emphasize relative spatial patterns of habitat suitability rather than absolute performance values and recommend spatial block cross-validation in future studies to provide a more conservative assessment of predictive performance. Third, the majority of species examined are from tropical and subtropical Asia, and incorporating temperate butterfly species could yield different niche dynamic patterns, though this was beyond the scope of the present study.

Fourth, niche specialization was not explicitly examined, as species with broad distributions may respond differently to climate change than range-restricted species; assessing niche breadth in relation to projected range changes remains a valuable direction for future investigation. Fifth, predicted range shifts for butterflies are also contingent on the persistence of their host plant species under future climate conditions, as butterflies are intimately dependent on specific plant communities for feeding and reproduction; disruption of these plant assemblages across Asian regions could constrain realized range shifts even where climatic conditions remain suitable. Sixth, although temperature and precipitation were used as the primary climatic drivers, the potential disruption of synchrony between climatic cues and photoperiod represents an additional unconsidered factor, as photoperiod is fixed by latitude and will not shift with climate change; butterfly populations moving toward higher latitudes may thus encounter photoperiodic conditions to which they are not evolutionarily adapted [40]. Seventh, binary habitat maps were generated using a fixed suitability threshold (0.6). Alternative thresholds would change the estimated extent of suitable habitat and projected range gains or losses; therefore, absolute range estimates are threshold-dependent.

## 6. Conclusions

This study provides a modeled perspective on how butterfly species across Asia may respond to climate change, revealing that despite projected climatic niche contractions in some species, the majority show potential for geographic range shifts under future climate scenarios. The modeled presence of niche unfilling and niche abandonment suggests that many species may retain some degree of ecological flexibility, potentially allowing populations to shift toward areas with newly suitable climatic conditions. However, it is important to emphasize that these projections reflect changes in climatic suitability alone and do not account for dispersal limitations, habitat fragmentation, host plant availability, biotic interactions, or physiological constraints that may prevent species from realizing projected range expansions in practice. The projected range expansions observed in this study should therefore not be interpreted as direct evidence of ecological resilience or enhanced adaptive capacity, but rather as model-based estimates of where suitable climatic conditions may emerge under future scenarios. While niche contraction remains a genuine conservation concern for a subset of species, the modeled potential for range shifts in others highlights the importance of incorporating multiple dimensions of niche dynamics when forecasting species responses to climate change. These findings underscore the value of integrating niche stability, expansion, unfilling, abandonment, and pioneering into conservation planning frameworks to ensure that management strategies account for the full complexity of projected distributional changes rather than relying solely on estimates of habitat loss.

## Figures and Tables

**Table 1:** Model validation metrics, including AUC, BOYCE, and IMAE generated using the MaxEnt algorithm for 200 butterflies in Asia. The standard deviations are presented in parentheses.

Species	AUC	BOYCE	IMAE
200 butterflies	0.92 (0.01)	0.92 (0.03)	0.87 (0.04)

**Table 2:** The average percentage of changes around the high suitability class under the climate change scenario in 2070 in Asia. The No. of Species Column (increase/decrease) represents the number of species whose distribution range will change under climate change scenarios. The numbers in parentheses also show the standard deviations.

Scenario	No. of Species (Increase)	Increase%	No. of Species (Decrease)	Decrease%
SSP245	185	37.6 (22)	15	11.4 (9)
SSP585	184	54.9 (31)	16	13.4 (11)

**Table 3:** Niche dynamics indices for 200 butterfly species under the climate change scenario SSP585 projected for 2070. Values represent the average percentage of raster cells corresponding to each niche dynamic category across all species, with standard deviations shown in parentheses.

Pioneering%	Expansion%	Stability%	Unfilling%	Abandonment%
1.1 (1)	12.1 (10)	62.8 (15)	15.7 (10)	8 (10)

## Data Availability

The Excel data and the list of species presented in this study are openly available at https://github.com/ehsanrahimi666/Asian-butterfliy.git, accessed on 20 April 2026.
